# Recurrent Focal Segmental Glomerulosclerosis: A Discrete Clinical Entity

**DOI:** 10.1155/2012/246128

**Published:** 2012-01-11

**Authors:** Elena Torban, Martin Bitzan, Paul Goodyer

**Affiliations:** ^1^Division of Nephrology, Department of Medicine, McGill University, Montreal, QC, Canada H3A 1A1; ^2^Division of Pediatric Nephrology, Department of Pediatrics, McGill University, Montreal, QC, Canada H3H 1P3

## Abstract

Focal segmental glomerulosclerosis refers to a set of particular histopathologic lesions in which steroid-resistant podocyte injury leads to patchy adhesions between the glomerular tuft and Bowman's capsule, followed by progressive glomerulosclerosis and proteinuric renal failure. Because of the nonspecific nature of this lesion, it has been difficult to classify the various forms of primary nephrotic syndrome in children. However, with the recognition of hereditary FSGS caused by mutations podocyte slit diaphragm genes, it is increasingly clear that the steroid-resistant form of FSGS that recurs in the renal allografts (R-FSGS) constitutes a distinct clinical entity. Capitalizing on recent studies in which patients have been screened for slit diaphragm gene mutations, this review focuses on the natural history and pathogenesis of R-FSGS.

## 1. R-FSGS in the Context of Steroid-Resistant Nephrotic Syndrome

A recent population-based study in the Gironde region of France reported an incidence of about 2.3 pediatric cases of idiopathic nephrotic syndrome for every 100,000 children <15 years of age [1]. The majority (91%) of these children exhibited a classical steroid-responsive relapsing disease, in which there may be podocyte foot process effacement noted on renal biopsy but no progressive renal insufficiency. Estimates of steroid-responsiveness are somewhat lower (80%) in referral populations or cohorts that include adults [2]. Among those who fail to respond to daily steroid therapy for 4 weeks, renal biopsies usually reveal a progressive lesion in which early podocyte detachment from the glomerular basement membrane is associated with segmental hyalinosis of the glomerular capillary tuft and, eventually, fibrotic adhesions to Bowman's capsule. It is the patchy distribution of these lesions within the glomerulus and the initial sparing of some glomeruli that warrant the pathologic descriptor, focal segmental glomerulosclerosis (FSGS).

In New York City, about two thirds of steroid-resistant nephrotic syndrome (SRNS) patients display FSGS on renal biopsy [3]. In the setting of SRNS, these lesions are associated with high risk of progressive renal failure [4]. Collectively, these children comprise nearly 15% of the dialysis and renal transplant population in North America. However, the FSGS lesion is not pathognomonic for a specific clinical entity but rather reflects any process that leads to patchy irreversible podocyte injury. In addition to the many disorders that cause “secondary” glomerulosclerosis, children with steroid-sensitive nephrotic syndrome (SSNS) may show mild FSGS lesions at presentation or later when biopsied for development of steroid resistance [5]. Yet, if steroid responsiveness is documented at any time, patients rarely have progressive renal insufficiency [6–11]. Conversely, some children with SRNS display only minimal histopathologic changes on initial biopsy, yet eventually develop end-stage renal disease. Thus, it has been difficult to dissect out the discrete clinical syndromes in children who present with idiopathic nephrotic syndrome in childhood. 

Among the SRNS patients who develop FSGS lesions and end-stage renal disease, about one third are now known to harbor mutations of genes encoding components of the podocyte slit diaphragm or adaptor proteins which link this structure to the podocyte cytoskeleton. These patients are generally unresponsive to immunosuppressive therapy but do very well after renal transplantation. Among children without pathogenic mutations of slit diaphragm genes, 30–50% exhibit recurrence of proteinuria and then gradually develop *de novo* FSGS lesions in the renal allograft. This phenomenon, first reported by Rich [12] and then Hoyer et al. [13], is generally taken as *prima facie* evidence for a circulating “FSGS factor” in the allograft recipient and serves as the signature for a distinct form of SRNS. Capitalizing on recent studies which screen out the most common gene mutations, this review focuses on the natural history and pathogenesis of steroid-resistant recurrent FSGS (R-FSGS).

## 2. Clinical Characteristics of Recurrent FSGS (R-FSGS)

Recurrence of FSGS in a subset of renal allografts has been extensively documented. However, until recently, it has been perplexing why some cases of primary FSGS recur while others do not. With broader screening for the recessive forms of genetic FSGS, the natural history of R-FSGS is now emerging with greater clarity. At one point, it was suggested that R-FSGS patients might differ from children with steroid-responsive nephrotic syndrome only in that the former carry a heterozygous mutation of a slit diaphragm gene. However, this does not seem to be the case [14]. In general, R-FSGS children have no identifiable mutations of slit diaphragm genes including nonpathogenic variants such as the R229Q polymorphism of the podocin gene [15, 16]. 

In 2010, Canaud reviewed 77 cases of idiopathic steroid-resistant nephrotic syndrome with FSGS who received a renal allograft [17]; the 42 patients who exhibited recurrent proteinuric disease had no demonstrable slit diaphragm gene mutations. Age at the time of presentation (10.4 ± 10 years) and delay before end-stage renal disease (4.8 ± 2.3 years) was similar to the group that showed no recurrence of proteinuria. At initial presentation, all were steroid resistant but 10/77 showed only minimal changes on renal biopsy. In the others, the FSGS lesion was subclassified according to 2004 “Columbia” criteria [18]; about half had a non-specific (NOS) lesion, 22.2% had the cellular variant, 12.9% showed collapsing lesions, and 7.8% had perihilar, and 7.8% had “tip lesions.” Thus, there is no distinctive pathologic subtype that distinguishes R-FSGS from forms of FSGS that do not recur after transplantation. Furthermore, as FSGS lesions gradually appeared in the allograft, less than 10% showed the same Columbia pattern as in the initial biopsy. In an earlier study of 19 R-FSGS patients, greater fidelity of the pathologic subtype before and after transplantation was reported [19], but it seems most likely that the glomerular histopathology reflects variability in the host response to podocyte injury or to modifications induced by treatment rather than a recognizable marker of the underlying etiology.

Clinical features of R-FSGS in the allograft roughly recapitulate the initial presentation. However, recurrence affords an opportunity to dissect the features of the earliest stage in detail. Heavy proteinuria may develop within hours of transplantation; this occurs in the absence of any FSGS lesions. Although proteinuria may rarely be delayed for several months (1/42), the majority in Canaud's study exhibited proteinuria within 48 hours (32/42) or within the first three weeks (9/42) [17]. About 90% of R-FSGS patients exhibit glomerulosclerosis when they first come to medical attention [17], but it is clear that FSGS lesions appear gradually in the allograft. Canaud identified FSGS in one patient by day 15 but documented the lesion in 28% of biopsies at 3 months and 38% by 12 months after transplant [17]. Appearance of FSGS lesions might have been more rapid in the absence of plasmapheresis and high-dose calcineurin inhibitors, but it seems that irreversible podocyte damage takes time and occurs well after the disruption of podocyte slit diaphragms that marks rapid onset of proteinuria.

## 3. The Putative Circulating Podocyte-Toxic Factor in R-FSGS

**Figure 1:**
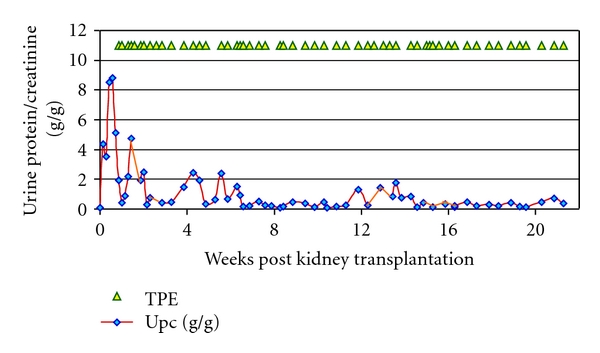
Immediate recurrence of FSGS (rFSGS) following deceased donor kidney transplantation. A 15-year-old girl with steroid-resistant FSGS had bilateral nephrectomy prior to transplantation. Proteinuria recurred within the first 12 hours but was controlled with intensive plasma exchange therapy (1.5 plasma volumes with albumin replacement). Efforts to wean plasmapheresis lead to a rise in urine protein/creatinine ratio (g/g) on several occasions. Each triangle represents a plasma exchange.

There is little doubt that patients with R-FSGS have acquired a circulating factor (or factors) that rapidly affects podocyte biology. Electron microscopy shows podocyte effacement at the time of recurrent proteinuria in the allograft and if patient plasma is applied to human podocytes *in vitro*, the cellular cytoskeleton is deranged within 6 hours [3, 20]. Sharma et al. argued that podocyte dysfunction in R-FSGS could be due to lack of a normal circulating factor, since replacement of FSGS plasma with normal plasma allows podocyte cytoskeleton recovery *in vitro* [21]. However, R-FSGS is transiently responsive to plasmapheresis [22]; this effect can be seen even when albumin (rather than fresh plasma) is used as the replacement fluid (Figure 1). Furthermore, Lagrue et al. reported the case of a woman, who had previously given birth to a normal child, and then developed SRNS with FSGS lesions [23]. In her two subsequent pregnancies, heavy proteinuria was evident in each newborn but resolved within 2-3 weeks [23]. This interesting observation shows that the FSGS factor can cross the placenta and suggests that podocytes can recover once exposure to the factor is ended. On the other hand, it also suggests that the circulating factor can persist for many days in the infant circulation or that podocytes need some time to recover. 

For many years, it has been assumed that the putative circulating factor is a cytokine derived from T-lymphocytes. This hypothesis was proposed by Shaloub in 1986, when he encountered a man with relapsing nephrotic syndrome and leukemia, involving a malignancy of natural killer-like T-lymphocytes bearing a chromosome 10 translocation; proteinuria resolved following successful chemotherapy of the malignancy [24]. Supporting this idea, Le Berre et al. reported that Buffalo/Mna mice develop a spontaneous form of R-FSGS which is ameliorated by infusion of CD4^+^CD25^+^FoxP3^+^ lymphocytes or by LF15-0195, a drug that increases the level of these cells in the circulation [25]. Similarly, Bao et al. found that R-FSGS, induced in mice by an antipodocyte antibody, is linked to a decay activating factor-dependent T-cell response [26]. However, recent reports have documented an effect of the CD20 B-cell antibody, rituximab, on relapsing steroid-dependent nephrotic syndrome [27–29], and Bagga described an effect of rituximab on steroid-resistant FSGS [30]. Thus, the time-honored assumption that human R-FSGS is a direct consequence of T- lymphocyte dysfunction must be reconsidered. 

Several groups have tried to identify circulating podocyte-toxic factors in patients with nephrotic syndrome. Savin and Sharma reported a 30–50 kDa factor in FSGS serum which appears to alter the permeability of isolated rat glomeruli to albumin, after exposure to 2% patient serum for 10 minutes [31, 32]. In this assay, the investigators measured the increase in glomerular volume that accompanies switch of culture medium from 5 g/L to 1 g/L albumin; unfortunately, it is difficult to know whether this assay reflects a change in permeability of the slit diaphragm or the capillary wall. In other reports, hemopexin, cardiotropin-like cytokine1, and soluble urokinase receptor have been proposed as potential candidates, but direct evidence for their involvement in R-FSGS is lacking to date. 

Most recently, Wei et al. reported that plasma levels of the soluble urokinase plasminogen activating receptor (suPAR) are elevated above an apparent threshold level (>3 ng/mL) in about two thirds of steroid-resistant FSGS patients and that suPAR induces proteinuria 24 hours after infusion into mice [33]. In elegant experiments with various suPAR mouse mutants, they found that this involves binding to and activation of *β*3integrin at the podocyte surface. In previous studies, they showed that *β*3integrin activation promotes cell mobility via small GTPases (Cdc42 and Rac1) that affect the cytoskeleton and showed that constitutive activation of *β*3integrin causes proteinuria [33]. These observations strongly implicate suPAR-induced *β*3integrin activation as a central mechanism causing proteinuria in FSGS. It is puzzling, however, why one third of their FSGS cohort had suPAR levels within the normal range (1–3 ng/mL). Conceivably, these patients have another circulating factor that stimulates local uPAR production by the podocyte or they may have some other, as yet unknown, circulating FSGS factor. Another paradox is that patients with chondrosarcoma produce elevated plasma levels of suPAR but uniformly do not develop proteinuria [34]. Wei et al. postulated that the suPAR released by chondrosarcoma cells may be functionally different due to alternative protein processing [33]. While some perplexing questions remain, activation of podocyte *β*3integrin by circulating suPAR may be central to the pathogenesis of R-FSGS. 

In 2009, Leroy et al. reported the case of a 12-year-old boy with R-FSGS in whom infusion of anti-TNF-alpha antibody induced rapid but transient complete remission. Remission was transient, but with each relapse, proteinuria resolved after infusion of etanercept, a synthetic fusion protein that blocks TNF alpha interaction with its receptor [35, 36]. In a preliminary report from the FONT study group, two of nine children with R-FSGS exhibited complete remission of proteinuria [35]. In an interesting case report, Assadi described a pregnant woman with HELLP syndrome (hemolysis, thrombocytopenia, elevated liver enzymes) and elevated circulating levels of TNF-alpha, whose newborn baby had nephrotic syndrome that resolved postnatally [37]; although the baby received hydrocortisone, the authors argue that transient proteinuria was most likely the result of transplacental TNF-alpha, since levels of the cytokine fell in parallel with resolution of proteinuria. TNF-alpha is expressed by lymphocytes and monocytes; podocytes express TNF-alpha R2 receptors and respond to cytokine stimulation by producing TNF-alpha themselves [38]. These observations suggest that in some cases of R-FSGS, TNF-alpha may constitute another circulating factor driving podocyte injury and raises some interesting questions about its relationship to the suPAR/*β*3integrin pathway.

## 4. Pathogenesis of Proteinuria and FSGS Lesions in the Affected Allograft 

**Figure 2:**
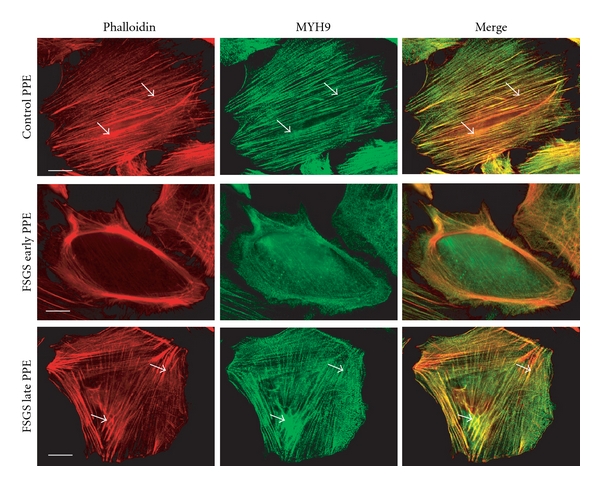
Plasmapheresis effluent from a patient with a recurrent FSGS disrupts the cytoskeleton of human podocytes in culture. Immortalized human podocytes (gift from Dr. Saleem) were incubated for 6 hours with 10% plasmapheresis effluent from a control patient undergoing plasmapheresis for a nonrenal disease (upper panel); PPE from a patient with recurrent FSGS collected at the start of procedure (middle panels, “early PPE”); end of plasmapheresis (lower panels, “late PPE”). The “early PPE,” but not the late PPE FSGS sample disrupts polymerized actin (phalliodin staining, red) and nonmuscle mysosin II (staining with anti-MYH9 antibody-green); arrowheads: actin stress fibers; scale bar 5 mc.

The effect of R-FSGS serum on the allograft glomerular filtration barrier is rapid and it is not uncommon to identify proteinuria in the immediate postoperative period [17]. Similarly, R-FSGS plasma disturbs the podocyte cytoskeleton within hours *in vitro* [39, 40] (Figure 2). These actin-based fibers are crucial for support of podocyte foot processes and, in biopsies performed shortly after transplantation, foot process effacement is seen by electron micrography. Furthermore, the actin cytoskeleton is linked indirectly via scaffold proteins to the slit diaphragm complex and, *in vitro*, R-FSGS plasma disperses nephrin from the slit diaphragms [20, 39, 41]. Interestingly, however, recurrence of proteinuria is sometimes delayed for weeks [17]. It is unclear whether late onset of recurrent proteinuria in the allograft represents a less aggressive form of the disease or whether primary progression of proteinuria was slower in such patients.

As noted above, there is often considerable delay between the onset of proteinuria and the gradual appearance of FSGS histopathologic lesions in the allograft. Current evidence supports the view that patchy irreversible injury to the glomerulus is preceded by podocyte detachment from the GBM [42–45]. FSGS plasma has been shown to disperse nonmuscle myosin from actin stress fibers *in vitro* [41]. Conceivably, loss of this contractile element in the podocyte cytoskeleton contributes to detachment by compromising cell contractility during pulsatile blood flow through the glomerular capillary. Another possibility is that podocyte detachment is disruption of focal adhesion complex proteins at the podocyte's basolateral surface. Chen reported that podocyte detachment and podocyturia is associated with decreased integrin expression in FSGS patients [16]. Babyeva has demonstrated rapid loss of podocyte focal adhesion complexes following exposure to R-FSGS plasma *in vitro* [2]. 

Although the severity of podocyte injury and detachment from the glomerular basement membrane may be driven by the same circulating factor that rapidly causes proteinuria in the allograft, it has been difficult to understand why histopathologic lesions appear only after months or years. Some insight into this paradox has recently come from two converging lines of investigation that assign an important role to the host response to podocyte injury in determining the long-term outcome. In 2005, Dijkman performed detailed analysis of a patient with R-FSGS, in whom Bowman's space and the glomerular tuft were “invaded” by parietal epithelial cells [46]. Several groups have provided strong support for the hypothesis that glomerular epithelial cells seen in the “proliferative” form of FSGS are derived from Bowman's capsule rather than arising through transdifferentiation of mature podocytes as had been proposed earlier [47–49]. The second set of important observations has come from Romagnani's group who have shown that mature kidneys retain a subset of renal progenitor cells in a putative stem cell niche at the urinary pole of Bowman's capsule. Their observations provide indirect evidence that these progenitor cells replace damaged podocytes throughout life. Taken together these two sets of observations raise the interesting possibility that FSGS lesions represent a disturbance of the normal process of podocyte replacement—occurring either when the system is overwhelmed by the magnitude of podocyte detachment or dysregulated by the circulating factor itself. In this view, proteinuria may reflect that immediate effect of the FSGS factor on podocytes, but the gradual appearance of glomerular lesions reflects insufficiency of the normal podocyte replacement process.

## 5. Treatment of R-FSGS

Although R-FSGS may be driven by a disorder of the immune system, the prospect of achieving sustained remission with immunosuppressive agents alone is limited. (a) Most R-FSGS patients were treated unsuccessfully with immunosuppressive agents (usually prednisone and calcineurin inhibitors ± cyclophosphamide for the primary disease in their native kidneys). (b) FSGS recurs in the allograft despite standard transplant immunosuppression. However, numerous groups have reported a rapid effect of plasmapheresis on proteinuria in some patients with R-FSGS [50]. When plasmapheresis is initiated shortly after disease recurrence, proteinuria improves substantially or resolves in up to three quarters of children; typically this involves three or four 1.5 x plasma volume exchanges per week for several weeks [51]. 

Unfortunately, many children who show excellent initial response to plasmapheresis recur as the frequency of treatments is weaned. In these children, a variety of sustained immunosuppressive regimens have been tried. Salomon reported rapid and sustained remission of R-FSGS in 30% of children treated with higher doses of calcineurin inhibitors (trough levels of 250–300 ng/mL for 3 weeks), proposing that this might overcome the effect of hyperlipidemia on downregulation of LDL receptors that mediate cellular uptake of the drug [52]. Remission was even higher (70%) among children who also received intensive plasmapheresis [52]. In a pilot study of adults with R-FSGS, Canaud used a combination of high dose (2 mg/kg by intravenous infusion for 2 weeks, plus oral cyclosporine to achieve 2-hour levels of 1200–1400 ng/mL, thereafter) and protracted plasmapheresis (1.5-plasma volume exchanges against albumin X3/week for three weeks with slowly decreasing frequency over nine months). With this protocol, sustained remission was achieved in nine of ten patients [22]. Dall'Amico et al. reported sustained remission in 7 of 11 patients treated with plasmapheresis and oral cyclophosphamide 2 mg/kg/day for 2-3 months [53]. Interestingly, among 30 reported cases of R-FSGS, about 50% have been reported to undergo sustained complete urinary remission following administration of one or two doses of rituximab 375 mg/m^2^ [54–56]. It is unclear whether the infusion of anti-CD20 antibody eliminates production of the circulating FSGS factor by B-cells, whether the depletion of B-cells indirectly alters T-cell function, or whether rituximab acts directly on podocytes [57].

## 6. Conclusion

About 30–50% of children with steroid-resistant nephrotic syndrome develop rapid recurrent proteinuria and thereafter develop slowly progressive FSGS in their allografts. Recurrent FSGS defines a distinct clinical entity involving a putative circulating factor that rapidly disrupts the podocyte slit diaphragm and then leads to irreversible podocyte injury. Until proven otherwise, it should be presumed that the pathogenesis of R-FSGS is distinct from steroid-sensitive idiopathic nephrotic syndrome and that heterozygous mutations of slit diaphragm genes have little impact on clinical features of the disease.
